# AR Enabled IoT for a Smart and Interactive Environment: A Survey and Future Directions

**DOI:** 10.3390/s19194330

**Published:** 2019-10-07

**Authors:** Dongsik Jo, Gerard Jounghyun Kim

**Affiliations:** 1 Department of Digital Contents Engineering, Wonkwang University, Jeonbuk 54538, Korea; dongsik1005@wku.ac.kr; 2 Department of Computer Science and Engineering, Korea University, Seoul 02841, Korea

**Keywords:** augmented reality, AR, Internet of Things, IoT, marker, markerless, tracking, interaction

## Abstract

Accompanying the advent of wireless networking and the Internet of Things (IoT), traditional augmented reality (AR) systems to visualize virtual 3D models of the real world are evolving into smart and interactive AR related to the context of things for physical objects. We propose the integration of AR and IoT in a complementary way, making AR scalable to cover objects everywhere with an acceptable level of performance and interacting with IoT in a more intuitive manner. We identify three key components for realizing such a synergistic integration: (1) distributed and object-centric data management (including for AR services); (2) IoT object-guided tracking; (3) seamless interaction and content interoperability. We survey the current state of these respective areas and herein discuss research on issues about realizing a future smart and interactive living environment.

## 1. Introduction

Recently, augmented reality (AR) and the Internet of Things (IoT) have received significant attention as key enabling technologies for making spaces smarter and more interactive [1,2,3,4]. AR is a type of interactive medium that provides a view of the real world augmented by spatially registering useful computer-generated information. It helps people to understand the world and amplifies their intelligence for solving problems and carrying out actual tasks [5,6]. IoT refers to a network of physical devices and everyday objects embedded with minimal computing elements for sensing, collecting, communicating, and even interacting with the objects themselves. Such an infrastructure will provide the basis for smart environments through a collective big data analysis and context-based services (e.g., real-time analytics and automation) [7,8,9,10].

AR and IoT might have different objectives with seemingly unrelated concepts, but they can, in fact, be complementary to each other [11,12]. First, AR offers a convenient and intuitive way for users to visualize and interact with IoT objects and their associated data. Principally, a spatially registered and visually augmented interface offers a direct and semi-tangible interface and is, thus, easy to comprehend and highly useful, particularly for everyday and/or anywhere usage [11]. The AR client, to wear a mobile or helmet-typed device, is capable of instantly connecting to an (IoT) product, receive relevant object specific data, control information and associated AR datasets for the given targeted service, then understand the state (or how to operate) with current datasets from the IoT product and interact with the physical object using direct control by natural interaction [11], for example. Note that semi-tangible means the interface that can be visualized and operated in a real-time way using the augmented virtual content to connect IoT objects in the real world [13]. Conversely, for AR, IoT as an infrastructure for “everywhere” service offers an efficient way to make AR “scalable” to the same degree by handling the necessary data management (for example, tracking data and content) in a distributed and object-centric fashion [1]. Thus, any IoT product can be accessed on the spot locally with the seamless manner, and the scalable interface allowed for location-based geographical and augmented reality services using AR clients [11]. Additionally, context-aware AR services are made possible by using and tapping into the more refined environment information made available by the IoT infrastructure [1].

To further promote this idea, that is, the synergistic marriage of AR and IoT, this paper surveys the current state of AR services in terms of their scalability and examines notable approaches to their integration into the IoT framework as a control interface. Here, the issue of scalability refers to the number of objects that can be supported without significant latency. Note that AR system performance depends largely on data management and object recognition and/or tracking. Additionally, to use AR as an interface with IoT, we examine previous studies that showed how AR can offer an intuitive and natural method to communicate with IoT objects, compared to other methods (such as using a graphical user interface (GUI) with no visual, contextual, or spatial registration). We believe that data management, object-guided tracking, and the interface design are key components for the development of an efficient IoT–AR infrastructure [11], and we focus on these three areas. To find out the current research status and future directions, first, we summarized previous works (See Table 1 for details).

To clearly highlight the current problems, as well as the requirements and potential benefits of the proposed idea, in Section 2, we start with examples of scenarios combining IoT with AR. Section 3, Section 4, Section 5 and Section 6 continue with a survey of these three key areas. We summarize the results and discuss future directions in the survey of these areas, as well as open issues for realizing a future augmented smart living environment. Finally, in Section 7, we provide some concluding remarks and a summary of our main findings and contributions.

## 2. Use Case Scenarios

**Figure 1:**
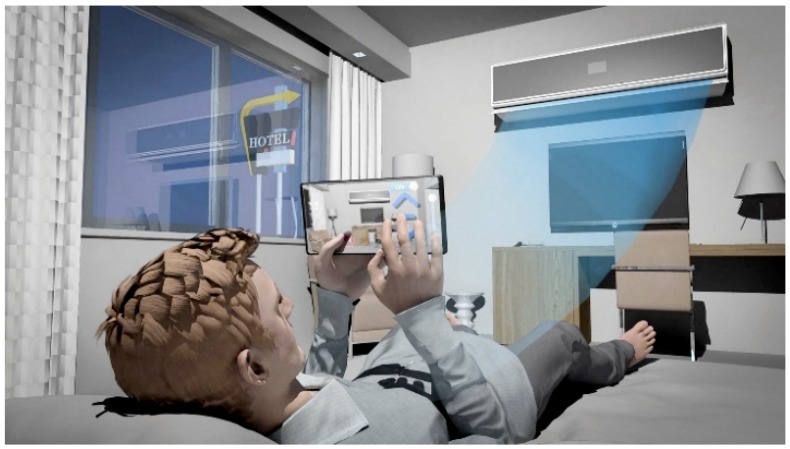
Use case scenario highlighting object-centric data management and intuitive interaction to enable “everywhere” augmented reality (AR) service through the Internet of Things (IoT) infrastructure.

John is on a business trip to New York. He feels a little chilly in the hotel and tries to raise the temperature of his room, however, he cannot find the thermostat. Using a standard AR enabled IoT service mobile app, he finds and connects to the IoT thermostat (using near-field communication such as Bluetooth), and the app shows an AR-based control interface with which John easily adjusts the temperature, without having to fiddle with the actual device or call the front desk for help (Figure 1). This scenario illustrates how AR services can be scaled to “everywhere” IoT-enabled objects. The AR client can connect to any IoT object using the assumed standard protocols through local peer-to-peer communication without having to use the central server through the Internet. Concerning the latter case, the central server would have to manage millions and millions of objects, with significant performance latency [11].

As another futuristic scenario in terms of AR tracking to connect IoT sensors, Alice wants to study the functionalities of her new TV and she is playing the AR manual for the TV. The AR manual provides step-by-step instructions to learn operations, and visual AR contents of next operation in the TV’s parts are registered with AR tracking information. Although Alice has prebuilt feature datasets, she cannot have robust AR tracking performance due to small feature points of the textureless TV. Thus, she pushes a graphical user interface (GUI) button for guided AR tracking, then she is able to execute robust registration selected by the TV’s tracking characteristic, which is the stored object’s tracking attribute in the IoT sensor. According to the described scenario, in the near future we will be able to apply object-optimized AR tracking to consider each object’s characteristics (e.g., rich texture or textureless).

**Figure 2:**
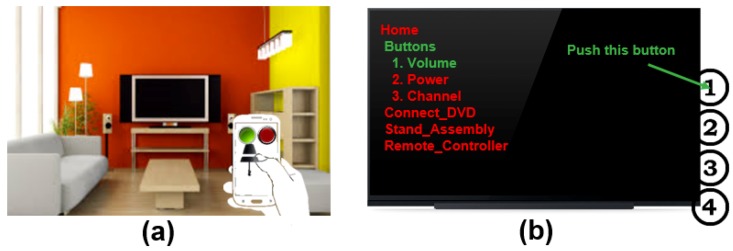
Augmented reality (AR) control for electronic systems: (**a**) physical light control through remote touch based on the AR platform [24]; (**b**) control status for the operating manual using AR visual information [1].

The final scenario is about augmented reality interaction. As an example of physical objects in everyday life, Richard is using his AR remote control system to turn on small appliances in his home [13,24] (see Figure 2a). He is in a spacious living room and decides to turn on a light that had been moved away from the TV yesterday, and he can control the selected light with the superimposed GUI button by operating the attached actuator. Note that AR interaction for home appliances refers to device control to exchange with the attached sensors and actuators in the real space. The techniques express the situations where the user can handle direct interactivity in terms of the physical devices. When the AR user stands near the television, for example, he can immediately obtain lots of useful status information from IoT sensors and directly control the TV to have desired control input using AR visual information [1] (see Figure 2b). Figure 2 shows an example of future AR control for IoT home appliances. Briefly, the AR user can execute direct interactions to manipulate all objects in the surrounding world.

## 3. AR Enabled IoT Platform for a Smart and Interactive Environment

**Figure 3:**
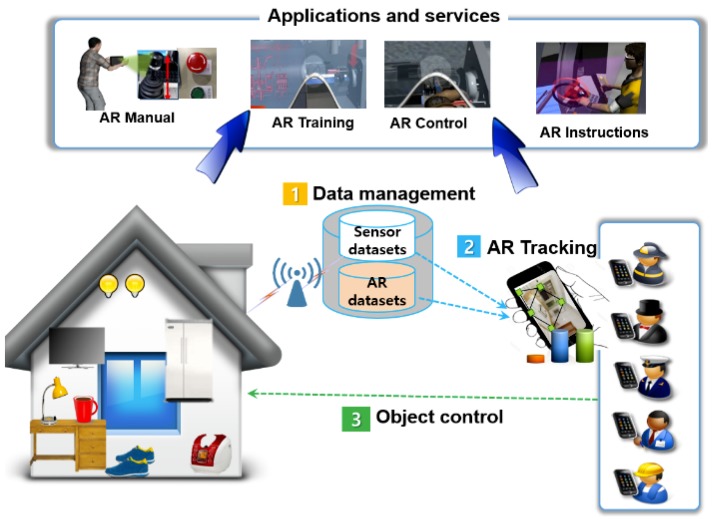
Internet of Things (IoT) combined with augmented reality (AR): overall possible architecture based on which smart and interactive AR services can be defined.

Here, we allude to three key components: distributed AR data management; object-guided tracking; context-based augmented reality (AR) interaction, and the resulting advantages of combining Internet of Things (IoT) and AR. Figure 3 shows a possible architecture of such combination as a basis for smart and interactive AR services. The AR service client interacts directly with the IoT object of interest in the immediate area and, upon connection, immediately receives context-relevant AR datasets (for tracking or customized service content) [1]. Depending on the context, appropriate and available services, such as a simple product information display, appliance control, or an instruction manual, are shown in the proper form (for example, through a mobile graphical user interface (GUI), AR glasses, mobile AR, voice, spatially registered AR, or simple overlay) with which to interact. 

Additionally, the AR system can track the relationship between a physical object and tracking information such as 3D features from images. Next, the user has the capability, in terms of efficient operation of IoT objects, to look through it with helpful AR content associated with the IoT environment. This is based on the help of both the IoT communication capability and the graphical AR environment. Then, any valuable application and service to connect everyday IoT objects and provide the user’s AR experience, such as an AR manual, training, control, and instructions, offers comprehensive platforms for ways that users can access and interact with mobile AR that cover everything and everyone to engage with the physical space [15]. Note that this typical approach can be realized using the filtering method that nearby AR clients to an IoT object can identify and augment the candidate target object to obtain contextual datasets [11].

**Figure 4:**
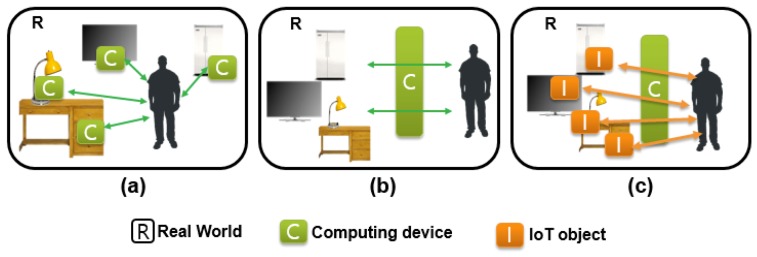
System combining augmented reality (AR) with IoT with a comparison of Human Computer Interaction (HCI) styles such as ubiquitous computers and augmented interactions [31]: (**a**) ubiquitous computer, (**b**) augmented interaction, (**c**) proposed AR mixed with IoT.

**Figure 5:**
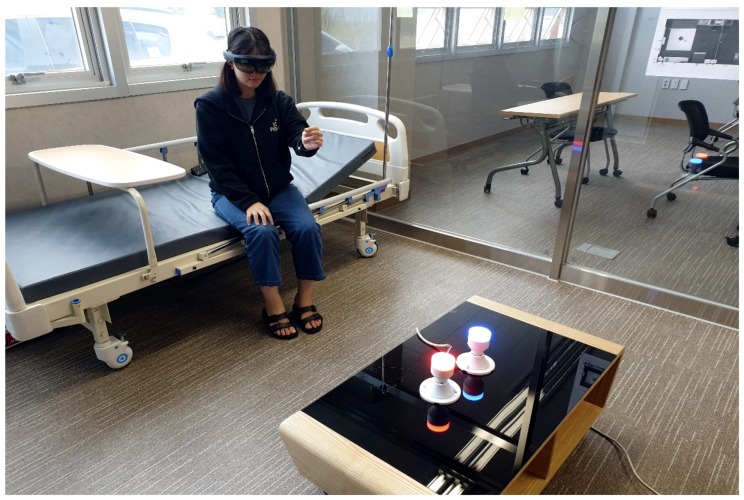
In situ operation of Internet of Things (IoT) lamps: an augmented reality (AR) user wearing a helmet-type AR device can interact intuitively (e.g., with hand gestures) to turn the lamp on or off with remote interaction, without having to fiddle with the actual device.

Figure 4 shows a system to combine AR and IoT in human–real-world interactions, with a comparison of ubiquitous computers and augmented interaction. Note that a ubiquitous computer is made to appear anytime and everywhere in contrast to desktop computing, augmented interaction refers to controlling in an AR environment, and the proposed AR mixed with IoT is to control the surrounding IoT devices using AR visualization and interaction. The mixing system of AR and IoT has the main difference that the user interacts with each IoT object embodied in the real world using computer-augmented information [27]. We can imagine that the system is formed by merging ubiquitous computers (Figure 4a) and augmented interactions (Figure 4b). Jo et al., for example, proposed an architecture for combining an AR interface with the IoT for shopping services. They developed a proof-of-concept prototype of the AR framework tested on IoT lamps and an in situ interaction method to support control directly with the IoT object (Figure 4c) [6]. Figure 5 shows the newly created system for our paper to explain in situ operation of IoT lamps. Here, an AR user wearing a helmet-type AR device can interact intuitively (e.g., with hand gestures) to turn the lamp on or off with remote interaction without having to fiddle with the actual device. We survey the state-of-the-art elements of these three key areas, in the next sections, to draw a clearer picture of what future IoT-enabled AR will look like, how it will operate, and what it will be capable of providing.

## 4. Data Management for Physical Objects

Augmented reality (AR) frameworks and services along with dataset management for everyday objects are described briefly in this section. Additionally, several architectures, data processes, data structures, and content representation for the physical objects to interact with AR in the published works are given. Internet of Things (IoT) and AR services commonly need to manage generic data and service content for their constituent objects or augmentation targets, respectively, which are physical everyday objects. Through having an IoT object communicate its own control interface information, the AR client can be configured to invoke certain control operations through AR interaction. Thus, the AR client has essential information to be contained in the IoT object, for example, features for AR recognition and tracking, generic content and information about the object itself, control interface, and organized additional contents for the operation. Note that the information exchange between the AR client and the IoT objects can occur directly between them or through the regional IoT server [1]. Herein, we review current approaches to managing such physical object data for AR use (for example, architecture and data handling) and discuss how they can be extended and scaled to the level of IoT [11].

To interact with real objects using AR interfaces in the early days, people were concerned with an AR framework that was capable of executing ubiquitous communication between physical objects and the AR device [31]. Recent works have attempted to deal with mapping the sensor–object relationship and filtering approaches (to reduce the search space) in the near space [1]. Specifically, in one notable framework with respect to AR, Iglesias et al. suggested an intelligent selection of resources by the user’s attributes, user–object proximity, relative orientation, resource visibility, and AR interaction connecting the object [32]. They developed an object browser based on AR with context-aware representation of resources. Additionally, Ajanki et al. constructed an augmented reality interface with contextual information and defined context-sensitive virtual information about people, offices, and artifacts [33]. Then, they suggested a filtered AR concept for finding out teaching and research project information to help a visitor to a university department.

**Figure 6:**
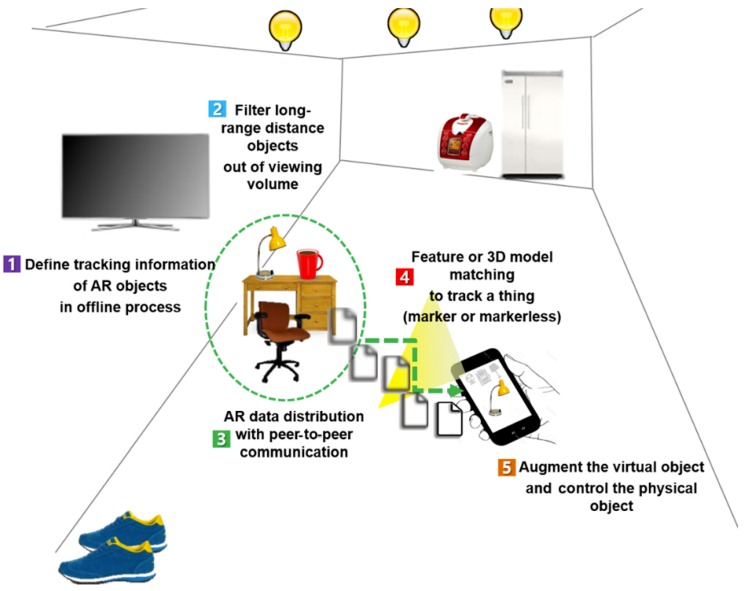
Future distributed data management scheme for “everywhere” augmented reality (AR) service for physical objects.

Figure 6 shows the future process flow for AR frameworks, based on the physical objects, considering AR datasets with scalability. An AR user interested in AR service can retrieve artificial markers (or natural features) from surrounding objects; it is necessary for the relationship between the physical and virtual object IDs to be defined in advance. Then, the user, holding the mobile device, is able to visualize the filtered AR objects, which are nearby IoT-capable objects based on their relative distance or direction from the user, in new places. Then, the client AR system directly receives the “feature” and “content” information for each object with the attached sensor. Next, the AR user can experience an efficient AR environment (e.g., IoT control interface) to mix the virtual object by donning a video see-through head-mounted display (HMD) or using a mobile phone with an attached camera module [1].

Many studies have been interested in augmented reality issues based on the cloud as the computing resource [34]. These works showed the benefit of computation time to reduce the heavy work by matching the large quantity of features for the poor computing capability of mobile devices. Additionally, recent works have focused on the process to register and manage AR datasets with a cloud computing device [34]. These works are concerned with how to fit tracking information and AR presentation datasets from the remote server. To access the surrounding objects, for example, the user can receive the collected AR attributes and tracking information that are shared via the server. Then, the AR user, with only the AR browser program without tracking information and AR presentation datasets in the device, easily can connect all of the things with the prebuilt relationship mapping. 

A more recent trend is to improve the way to use the adjacent computing resources in the user’s surroundings, rather than enabling computing services on the end of the network at a long distance, such as a remote server or the cloud [1]. It will still be difficult for cloud services to support scalability to the level of “everywhere.” An alternative may be to connect to a single area server (serving only a particular local area, such as a single home) managing only a limited number of objects [34]. The adjacent computing approach can be used to solve problems such as a bottleneck assignment and detection of moving objects by a remote server. Note that this approach is similar to fog computing architecture in the domain of a sensor network that emphasizes latency reduction with high-quality service and handles datasets at the network edge [16,17,35]. The AR user can connect directly to the objects in the surrounding area because the sensors attached to the object can detect it in real time to consider the user’s position in certain ranges [17].

**Figure 7:**
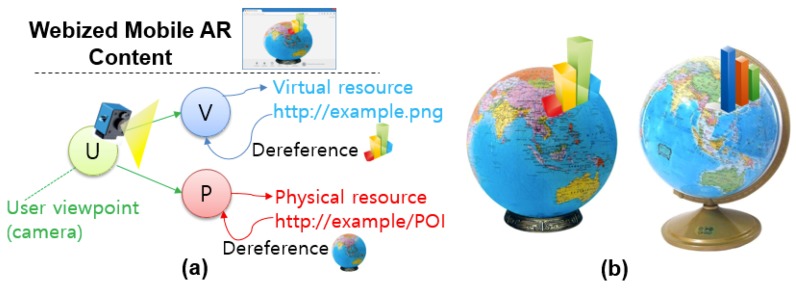
Webized augmented reality (AR) content representation in which virtual data are associated with a Web-accessible physical resource [37]: (**a**) virtual and physical resources of webized AR content, and (**b**) an example associating a physical sensor dataset [38].

Some research has proposed details of the content structure for resource management as another issue to provide AR datasets. Kim et al. presented an AR content structure for building mobile AR applications in HTML5, as on the Web [28]. They used an extended presentation logic of HTML to apply current web architecture and a referencing method with matching between physical and virtual resources. To validate their process, they augmented a physical globe with sensor data fed from physical weather sensor stations (see Figure 7). Additionally, as a similar AR data structure, Muller et al. introduced a custom XML-based format to define AR manual structures for home appliances [36]. 

The situation is similar with IoT services and content. Most IoT services are implemented as applications. One promising direction is to use the Web to support interactions with physical objects, as exemplified by Google’s Physical Web [37]. Here, objects have URLs and can exhibit their own dynamic and cross-platform contents, represented in standard languages such as HTML and JavaScript. Thus, we can envision a future where various types of IoT services, including even AR, will be available under a unified Web framework, that is, the “webization” of things. Ahn and co-workers, for example, presented a content structure as an extension to HTML5 for building webized mobile AR applications [18]. This allows a physical object to be referenced and associated with its virtual counterpart. Figure 7 shows an example of associating a globe (physical object) with virtual objects (augmentation).

Additionally, we should consider the characteristics of AR contents according to IoT devices because there are many different types of IoT devices. Note that this is a similar concept that the website components have the different configuration in mobile and desktop computing devices. Thus, to make the IoT enabled AR platform to be naturally applied everywhere, depending on the nature of the IoT device, it should adaptively control the degree of AR content representation.

## 5. Scalable AR Recognition and Tracking for “Every” IoT Object

Augmented reality (AR) research primarily focuses on overlaying a virtual 3D model, that is, on how to realistically integrate augmentation with the real world. Recently, AR technologies related to the context of physical objects have increased [22]. These are related closely to intuitive visualization with attributes that can help the physical objects easily interact with the surrounding area. “Mediating Mediums” by Greg Tran describes an AR 3D architecture system based on contextual relationships with real geometry for interrelationships between digital and physical objects [39]. He suggested a system to provide simulated geometries projected into videos of physical space. Plus, there are many works on AR tracking methods involved in analyzing useful sensing information with the main focus of prompt decisions of daily Internet of Things (IoT) objects. Claros et al. proposed a fiducial marker-based AR medical system to monitor real-time information with collected biometric signals from patients [40]. They used a wireless sensor network (WSN) to process semantic information collected from distributed sensors, and a marker ID overlapped with the real world to visualize perceptual information (e.g., temperature and humidity). Considering another approach, Mihara et al. implemented a light-emitting diode (LED) AR marker with the procedure of reading LED blink patterns attached to a TV rather than fiducial markers [29]. However, the biggest problem of predefined fiducial markers and pattern IDs is that they must have individual markers corresponding to each physical object. Therefore, if there are many objects in space around the AR user, huge amounts of markers corresponding to the number will be needed, and large amounts of feature datasets for tracking must be made beforehand. Thus, an AR system based on natural feature tracking would be more effective to improve the tracking performance due to helpful information associated with the natural characteristics.

Fast, correct, and stable recognition and spatial tracking (that is, pose estimation) of objects represent the most important technical challenges for AR. Another recently popular technique is a feature-based method that identifies an object and computes its pose using primitive geometric features and their properties detected by a sensor [41]. Different feature-based techniques vary in their robustness and may be applicable to certain classes of objects. Feature-based methods also require a relatively high number of features (and data) to establish a robust match and often require a preliminary learning phase to handle difficult matching conditions (for example, an angled view, dark lighting, occlusions) [21].

**Figure 8:**
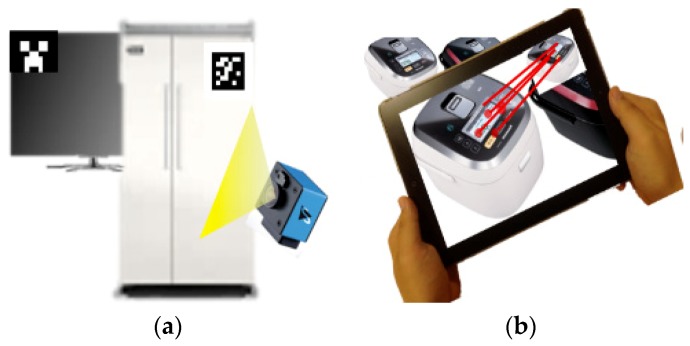

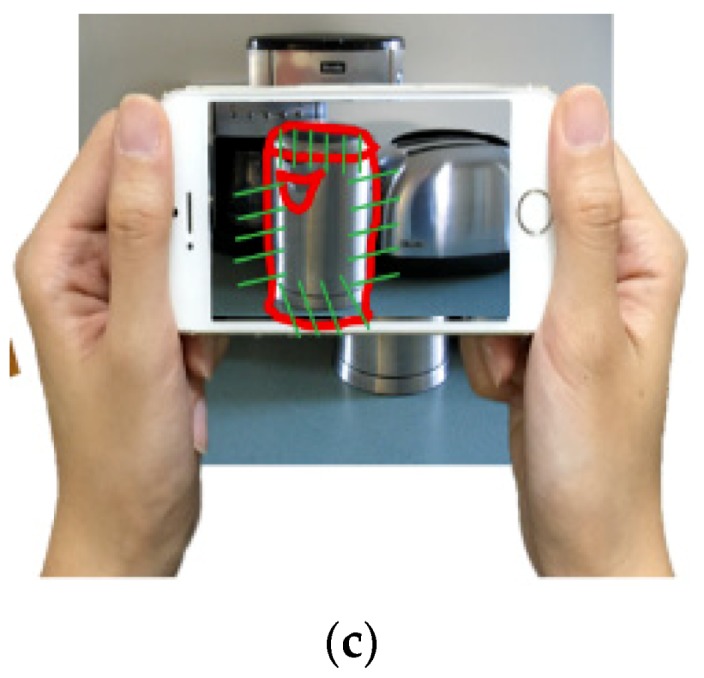
Main object recognition and tracking solutions for augmented reality (AR): (**a**) marker/fiducial, (**b**) feature, and (**c**) model-based.

Regarding objects without rich features (for example, textureless objects), template image matching is used often; however, this method has many disadvantages for use in robust 3D tracking [33]. A few new approaches to the problem of tracking textureless 3D objects have been developed for situations such as under poor lighting conditions, during partial occlusions, and against cluttered backgrounds, but they remain very difficult [21]. Model-based tracking that attempts to recognize and track target objects by matching and fitting a 3D wireframe model to the edges extracted from a camera image has been proposed [42] (see Figure 8). However, such model-based approaches usually require a good initial solution with a lengthy convergence time, and it is not clear which reference 3D wireframe model would be the most suitable. Despite such complications, they remain viable alternatives, given the absence or lack of feature information.

Thus, scaling such methods for millions of IoT objects would be even more difficult (either the level of accuracy or the capability of real-time response is likely to suffer). Placing and attaching markers to thousands of everyday objects also is not a practical solution. Additionally, because there is no single universal recognition and tracking method to cover all types of objects, a multitude of algorithms can be used collectively. Therefore, in a typical situation, before the object is recognized, one cannot determine a priori which algorithm would be the best to apply to its recognition in the first place. Plus, all algorithms must be attempted exhaustively, which will again result in significant latency.

## 6. IoT Object Control with Scalable AR Interaction

**Figure 9:**
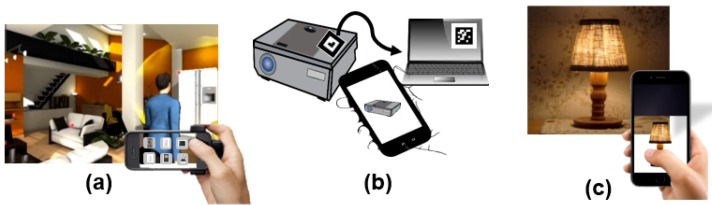
Various augmented reality (AR) interactions with Internet of Things (IoT) objects: (**a**) in situ/remote operation with traditional graphical user interface (GUI) button; (**b**) metaphorical natural interaction (virtual dragging) to invoke an object function [45]; (**c**) interacting in a virtual/augmented space to affect the physical world [11].

The most prevalent application of Internet of Things (IoT) currently involves object control, either remotely or in situ. IoT objects consist of sensors for incoming data, networking modules for wireless capability, and actuators to control the object’s functionality [42]. Expressly, actuators can operate with the user’s interaction, such as the decision of object properties (e.g., turn on lights). Jackson et al. showed the operation method of the object’s behaviors (e.g., TV) with a simulated environment for home automation [25] (Figure 9a). More recently, a few research papers have shown visualization of interconnected simulation results with a sensor (or actuator) embedded in an everyday environment. Lifton and Paradiso presented a dual reality system that generates an interplay between the simulated world and a bunch of sensors, such as an electrical power strip in the real world [43]. Users can explore a variety of experiences in the simulated dual reality; this concerns not only mutually reflected sensor browsing but, also, interaction techniques with the interconnected sensor through sensor/actuator networking. Lu proposed a bidirectional mapping technique for IoT-enhanced information visualization [30]. When a user turns on an appliance (e.g., TV) in the real environment, for example, the attributes of the deployed sensor to detect the user’s activity are transmitted to the simulated world. Then, the monitored virtual world can generate a counterpart representation in the real world. This system was developed to realize eco-feedback for energy saving [30].

Some research in the augmented reality (AR) field is about simulation related to the control of the object’s function and usability measurement [44]. AR also offers an excellent method of in situ object control (even for remote objects, using a remote-controlled camera) [1]. AR can be used to visualize simulations of applied control for previewing or training purposes [44], for example. 

Recently, there have been a few attempts to merge the two, for example, using AR (or even virtual reality (VR) as the control and simulation interface for IoT objects. Moreover, people have recently tried direct control for everyday objects in the AR environment. Rekimoto and Ayatsuka proposed a visual tagging system called CyberCode, which is based on an AR 2D barcode to identify and detect objects [45]. The system has an operation mode to manipulate physical objects. Subsequent to selecting a manipulating object, for example, the user performs a “drag-and-drop” to another target object with natural interaction (e.g., the notebook in Figure 9b). Then, the target object would carry out the particular operation with useful information in the first selected manipulating object (e.g., retrieve a currently displayed slide image). Another example would be Muller et al. suggesting an AR manual to convey step-by-step instructions. They defined a user markup manual language (UMML) file to generate sequential operations with corresponding steps. Consequently, in the near future, IoT objects will connect with each other and AR users in the operation environments will be able to intuitively manipulate the context of objects, receiving help from AR technologies (IEEE Consumer Electronics Magazine) (Figure 9c).

What is more, the actual user experience will depend vastly on the AR device. Kruijff el al. provided a classification of perceptual issues in augmented reality and suggested predominant issues for a specific device (e.g., Head-worn display, handheld mobile device, projector-camera system) [19]. Most previous works mainly have used smart phones to provide images that synthesize real and virtual environments, but they did not consider the presentation of synthesized images directly to the human eye. More recently, AR devices with a helmet-type head-mounted display (HMD) (or head-worn display) that synthesizes spatially registered virtual objects overlaying a user’s view have been introduced. These helmet-typed AR devices are mainly divided into optical and video see-through HMDs, depending on whether actual images are viewed directly by the user or via a video input. Note that the video see-through head-mounted display (HMD) allows a dual-webcam module to be attached to an immersive HMD display to have two image sources, i.e., the real world and the computer-generated world. Alternately, the optical see-through HMD has the capability of mixing virtual objects to allow the user to see through them, i.e., the computer-generated world [46]. Therefore, we are interested a framework for AR interaction considering the object characteristics for interacting IoT objects and the impact of various kinds of AR devices.

## 7. Future Research Directions

### 7.1. Proposal: Data Distribution and Peer-to-Peer Communication

Most previous works had difficulty dealing with the representation of the augmented reality (AR) object for users to access a new place that they had not visited [11]. Previous related works in the AR research field handled the stored data by precalculated tracking information (e.g., feature sets by known fiducial markers) rather than online data loading., The Physical Web to connect any smart device (or object) carrying a Web address was developed [37] as one of the near future AR scenarios. This is a data exchange approach for Internet of Things (IoT) objects to provide interaction on demand with direct sensor access related to all things in surrounding areas. Sensors based on high-speed networking, as a similar approach to apply AR environments, allow users in the augmented reality environment to immediately perceive natural AR experiences with the communication method of AR datasets used for recognizing and tracking them anyplace and anytime. To augment a target object containing AR information, the AR system expects standard data formats such as feature sets, augmentation contents, and control interface [1]. Thus, AR visualization and operation in the applications for IoT objects will require a standard formatted structure for 3D object attributes (e.g., function, permission, behavioral animation, and filtering protocol). Additionally, an AR visualization and interaction environment for IoT services (e.g., social interaction) is based on standard event handling mechanisms for cross-platform compatibility with popular Web browsers (e.g., WebGL) [47].

Furthermore, IoT datasets can be very large for processing when data are generated over a certain amount of time [11]. Thus, future research will be carried out on methods for efficient AR presentation of visual information on real-time big data analytics (e.g., AR object size, shape, population density). Then, an AR technique supported by IoT will expand for development, with the purpose of object filtering to consider contextual information in the user’s surrounding area for AR services between the object and the user. Taking this user-centric AR filtering approach, the AR consumer easily can connect a small number of things with the scanning target [1]. 

Presenting another issue, in complex situations, to handle a lot of everyday IoT objects with huge amounts of IoT devices in the surrounding environment, AR augmentation content needs to define and optimize the representation complexity of a virtual object related to sensing information of the IoT objects according to the viewpoint-relative position. The optimized level of detail (LOD) technique is helpful to increase the efficiency of rendering by decreasing the workload on an AR computing device. 

The performance problem in a scaled environment such as IoT is manifested by the amount of time needed to look up and match the target object and handle and/or process associated data and/or content among millions of candidate objects through the network. Allowing for IoT objects having their own computational, networking, and storage capabilities, data and/or content can be distributed, stored, and exchanged (even without the Internet infrastructure). Instead, IoT objects themselves can communicate the necessary data to clients on a need-to-know basis (including the information required for their recognition and tracking). That is, the data are now delegated and distributed to individual objects in the environment. Such a scheme has the added advantage of faster and more robust object recognition and tracking. The proposal is depicted in Figure 6. 

The AR client in Figure 6 detects the presence of IoT objects (equipped with elementary processing, storage, and network modules) in its vicinity (similar to identifying Wi-Fi access points) [1], and these objects communicate the necessary tracking information to the client. Since there is bound to be relatively few target objects around, the client quickly can identify (and even track) the objects and retrieve the associated content. Note that neither a server nor the Internet is involved in this realization. It could be argued that the IoT objects simply need to be organized geographically and managed through a hierarchical network of servers (similar to a geographic service). However, if the enormous number of objects to be handled (even compared to that of geographic objects) is disregarded, there is currently no common technology for accurately tracking and recognizing individual objects (which may be mobile) in indoor locations. Therefore, IoT provides an ideal and natural infrastructure for “everywhere” interaction with physical objects. Note that the data and/or content also can be uploaded to the objects to add and create new IoT services and applications.

### 7.2. Proposal: Object-Centric Guided Tracking

**Figure 10:**
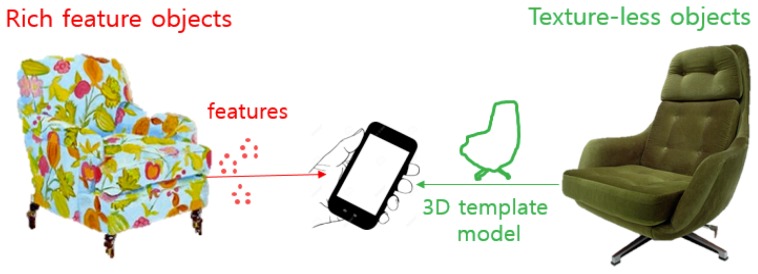
Guided AR tracking considering object characteristics [1].

The proposal described in the previous section on data and/or content distribution to Internet of Things (IoT) objects can also be used to easily solve this problem (scaling of recognition and tracking for augmented reality (AR). That is, in addition to generic data and service content, individual IoT objects of interest in the vicinity of the AR client can communicate the information required to recognize and track itself, including features, algorithm type, and even physical condition (for example, lighting, distance, or another companion reference object). The AR client is “guided” by the target object itself to localize and track it [1]. Note that in this scheme, the number of candidates in the matching is relatively low, consisting of only those candidates in the interaction space of the AR client or user. This, in turn, makes it feasible to use a collective algorithmic method and reduce the number of features, templates, and models in the matching process, further lowering the data requirement. Figure 10 illustrates an example of guided AR tracking in which objects with different characteristics can be localized and tracked quickly and robustly (for example, feature-based tracking for rich feature objects on the left, and template-model based tracking for textureless objects on the right).

**Figure 11:**
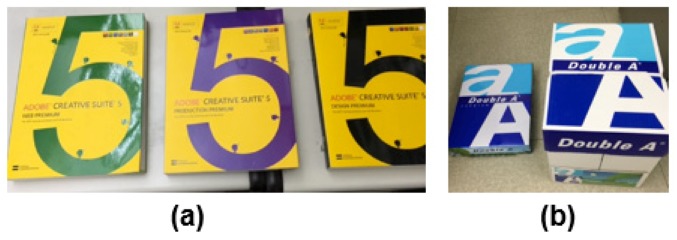
Examples of complex situations with similar shapes or the same feature sets in surrounding areas: (**a**) same shapes and different-colored textures (**b**) same textures and different shapes.

Soon, according to high-speed networking and anywhere communication, users will be able to track virtual objects that have direct communication with IoT objects [45]. Therefore, future research will be directed toward developing new AR tracking to overcome complex situations for a lot of everyday IoT objects. Then, IoT technologies will be helpful to improve the robust AR tracking performance. Since the physical object needs to have its properties already known in the approach for robust AR tracking, for example, we can apply a guided tracking approach for potential target objects by the decided tracking method using the object’s characteristics in advance. Additionally, when choosing an AR tracking method in terms of a target object, we can consider things that have a similar shape or the same feature sets in the complex IoT environment (see Figure 11).

### 7.3. Proposal: AR Interaction Framework for Object Class

**Figure 12:**
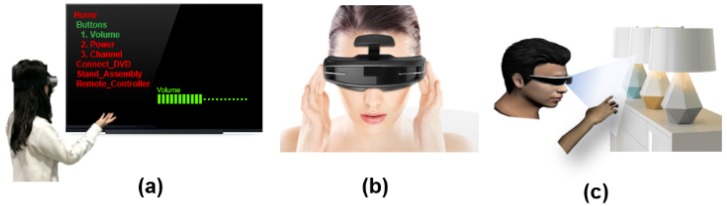
Different styles of AR interaction using object type and requirements. Such information is obtained directly from the object, similar to the case of tracking information: (**a**) natural gesture used to carry over a task such as controlling TV volume; (**b**) swiping interaction for an AR manual searching task; (**c**) pointing gesture for toggle interaction, such as operating a lamp’s on/off switch.

Regarding the IoT, AR-based interaction and object control have been shown to be much more intuitive, direct, and helpful compared to graphical user interface (GUI) - and menu-based interfaces on handheld devices, for example. GUI- and menu-based interfaces still have the advantage of consistent design and guaranteed minimum usability [28]. Thus, our proposal is to develop a framework for AR interaction considering the object characteristics and the impact of various kinds of AR devices. Again, similar to the concept of guided tracking, such information can be obtained directly from the object and a particular interface can be tailored adaptively to objects given the client platform. Simulating an action to open a printer for maintenance purposes, for example, might be carried out through a natural gesture (allowing the user to have a better understanding of how to carry over the task to the real world), whereas a GUI and/or menu will suffice for simple option selection. Interacting with certain objects might require an overlaid view and, as such, augmentation should be placed with an offset to the object or overlaid with transparency [40]. The IoT infrastructure offers a way to customize the interaction method and style, however, customization at the level of each individual object or object function will be a daunting task. A classification of objects and object functions, and a design framework to map them to various styles of AR interaction, will be required. AR-based object control with intuitive visualization of the object and direct response feedback to operate (e.g., turn on a light) will be helpful in interaction and control of the object rather than previous GUI button menu-based operation. Therefore, future research on aspects such as operating usability and interface will need to reflect the context of the environment (See Figure 12) and the user experience for natural control interface to manipulate IoT objects [48,49,50]. An AR user interface providing bare-hand operation to manipulate in the situation of wearing an head-mounted display (HMD), for example, will be developed continually [51,52].

**Figure 13:**
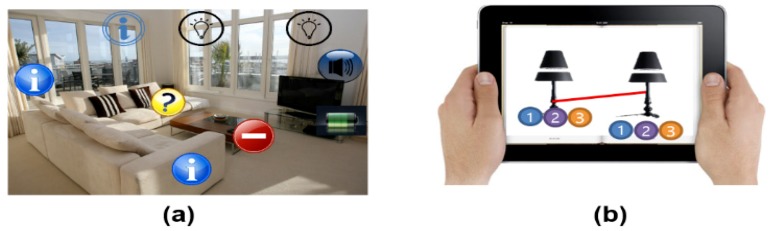
Augmented reality (AR) approach for interaction of everyday IoT objects: (**a**) interactive tool to define the operation of indoor IoT objects by hierarchical functional grouping [24]; (**b**) AR system to define the behavior of objects to create new functionalities [20].

Additionally, to develop specific applications enabled by the IoT infrastructure, we need to abstract the object functionality with a set of sensors. Usually, it is based on works dealing with IoT middleware. Specifically, the visual programming of sensors and actuators allows the user to direct operation [53]. Figure 13 shows a definition process of higher-level information with the raw data collected from the sensors of physical objects [24], and an interface to define relationships among physical objects and create new functionalities that enable an object‘s behaviors [20].

## 8. Conclusions

Recently, smart Internet of Things (IoT) users will operate and interact with physical objects that can receive augmented reality (AR) datasets anytime and anywhere and can enrich their visual perception. We surveyed various AR approaches (e.g., AR tracking, interactivity) to enhance the usability of physical IoT objects in the user’s surrounding area and provided the classification and taxonomy for IoT enabled AR. Expressly, we summarized the recently developed data management techniques to handle AR datasets connecting everyday objects, AR tracking for everyday objects, and direct control-based AR interaction for physical things categorized as the three key components. Moreover, we reviewed recent AR applications and suggested future scenarios related to IoT objects. Additionally, we suggested the open issues that require further research to enhance the user’s AR experience for highly connected IoT objects in the surrounding environment. We described how the current bare IoT infrastructure can be extended to include smarter and more effective user interactions. Individual or meaningful sets and groups of IoT objects can be imbued with data and/or content in a distributed manner and efficiently utilized by the client. The distribution makes it possible to scale and customize interaction techniques such as AR. Table 2 summarizes the current status of AR and IoT separately, along with the potential advantages and expected synergies of integrating them.

We plan to demonstrate our proposal in the future, using an actual prototype system and validate our claims in terms of its improved usability and performance. We plan to continue to upgrade our survey with different system architectures and research issues in accordance with display types such as mobile phones, wearable glasses, and projection-based spatial AR. Additionally, we will summarize recent approach with an IoT-centric survey to add the proposed AR-centric approach in the environment to combine AR and IoT.

## Figures and Tables

**Table 1:** Classification of Issues and Problems in AR and IoT.

Issues	Problems		References
Data management	AR	prebuilt 3D virtual object datasets, prestored AR content	Jo et al. [11]
		context-aware having accurate information about the surrounding environment	Jo et al. [1], Jo et al. [11], Suh el al. [14]
	IoT	access to distributed sensor data (filtering), scalability (IoT perception)	Michalakis et al. [15], Bonomi el al. [16], Jesudian et al. [17]
		quality of service, customize the system according to the user’s needs	White el al. [2], Michalakis el al. [15], Gimenez et al. [4], Ahn et al. [18]
		object relationships, exchange of resources among objects	Kruijiff et al. [19], Campana et al. [20], Kasahara el al. [13]
		law interoperability	White et al. [2]
Viewer and display device	AR	AR registration ambiguity, registration errors significant latency, augmentation method, occlusion, tracking for the dark environment field of view, the need for abundant markers	Kruijff et al. [19], Michalakis et al. [15], Diao et al. [21], Newman et al. [22], Negara et al. [23]
		predominant viewing by the specific AR device (e.g., head-worn display, handheld mobile device, projector–camera system)	Kruijiff et al. [19]
	IoT	unintuitive context information, undirected viewing	Michalakis et al. [15]
		result display with physical characteristics	Phupattanasilp et al. [3]
		authoring IoT	Jeong et al. [24], Jackson et al. [25], Heun et al. [26]
Interfaces and interaction methods	AR	fixed interaction method	Choi et al. [27], Kim et al. [28]
	IoT	undirected interaction, limitations on intuitively communicating services	Jo et al. [11], Mihara et al. [29], Lu et al. [30]
		response time of the application	White et al. [2]

**Table 2:** Prospective features from combining AR and IoT.

Key Components	Generic Features of AR and IoT		Potential Features to Combine AR with IoT in the Future
AR data management	AR	Visualization with prebuilt 3D virtual object datasets and prestored AR content including a substantial amount of storage	Scalable AR dataset management and services able to directly access and immediately exchange contextual information of everyday objects for efficient AR representation Context-aware service having accurate information about surrounding environment (filtering) Customize AR systems according to the user’s needs in terms of IoT devices Exchange of resources and datasets among IoT objects
	IoT	Descriptions of and access to distributed sensor data (for example, context information)	
Object-guided tracking	AR	AR registration in only one way (for example, feature detection or model-based)	AR tracking methods based on binding between real space and virtual object to be superimposed over real IoT object in real time Robust object-optimized tracking by considering tracking characteristics of each object Accessorial tracking information in limited environments (e.g., dark lights) Intuitive visualization with physical characteristics of IoT devices AR authoring for IoT devices
	IoT	Resource monitoring collected from objects that are embedded with sensors (this is not considered virtual imagery to be superimposed on a real IoT object)	
AR-based object control and interface	AR	Interaction to manipulate virtual objects through a fixed method	Object class-wise interaction customization and optimization Intuitive interface to manipulate with AR interaction viewing direct response in terms of functionality of physical objects
	IoT	Control used to operate IoT sensors with an indirect viewing interface (for example, GUI menu-based IoT object control)	
